# Editorial

**Published:** 1874-10

**Authors:** 


### Editorial.

***Sponges.***

We are all familiar with the qualities of that useful article, the sponge, its active absorption and ready yielding of its contents—qualities which stand exactly correlated, so that the sponge gives forth from its tissues when required all, even to the last drop, which it had absorbed. These sponges are useful only when dead.

There is, however, another species of the genus sponge, whose functions, most actively performed in the living state, are somewhat different from those of the first, for they appear to possess only that of absorption, or suction, rather, by which they extract all that is possible from those to whom they attach themselves. What they have absorbed, they yield reluctantly, and then only by the principle of substitution, by which they may displace their contents by something more valuable.

They suggest very forcibly that they may have been one of the sources from which Victor Hugo derived the inspiration which was developed by his genius into that wonderful word-picture, "*La Pieuvre*" in *Les Travailleurs de la Mer*. That one sucked the blood of its victim; this one sucks that highest elaboration of the blood, his brains.

Many of our older readers will recognize this being from what has been already said of him; but, for the benefit of the younger members, we will describe his *modus operandi.*

He enters your office with a "smile that is child-like and bland," and a compliment like a sugar-coated pill of aloes—sweet without, nauseous within. He is modest, too, this sponge of ours. He does not ask your purse; he does not even imitate that other fiend, the book-borrower, who, having only money enough for whisky and cigars, cannot buy books, but borrows from his more frugal neighbor, and proves himself always that paradox, "A poor accountant, but good book-keeper." Our sponge only comes to borrow a few ideas; in fact, he would like the use of your brains for an hour, and although they may be busily occupied in earning bread for yourself and family, he cannot wait; he must get to his office by his office hour.

He has a very doubtful case; has made no diagnosis; has called into consultation the learned Professor A. or B., who has agreed with him entirely, and—taken his fee. But the case halts, and now he stops to administer the sugar-coated pill, "knowing that you have paid special attention to such subjects," "he would like your views"—which you are generally weak enough to give—and, with many thanks, and a teeming brain, the *sponge* retires, treats his patient successfully, with modest complacency accepts the credit and the amount of his bill, perhaps publishes his case, giving about ten dollars' worth of the former to the consultants, Professors A. or B., and ignores entirely the source of his ideas.

Shakespeare said, "There be land-rats and water-rats, land-thieves and water-thieves;" and a higher specialization of these would define those who steal your goods, and your reputation; but even he, myriad-minded as he was, had no conception of the thief of brains, the intellectual confidence operator, who boldly borrows your ideas to carry him through his difficulties, and hands you, in return, his bogus check, flattery and modest assurance.

This evil is serious. The practice deserves the denunciation and execration of all who respect themselves and their profession. There are physicians in this city who are daily subjected to this sort of extortion, either by personal application or by letter, from men who, too ignorant or too indolent to form original opinions, and lacking the moral courage to acknowledge their own deficiencies to their patients, and to demand consultation—from which some other might derive professional and pecuniary benefit—meanly prefer to borrow the lion's skin wherewith to clothe the jackass. H.

The Philadelphia *Medical Times* thinks that the Commissioners of Charities and Correction in New York must have been influenced by the most weighty reasons in removing from their position on the staff of Bellevue Hospital, twelve of its nineteen members. We should think so, too, but would, in common with the majority of the profession of the whole world, be much gratified to know what those "weighty reasons" may be which are deemed sufficient to disqualify men like Hamilton, Flint, and their associates, to hold positions which, by the universal judgment of their own profession—best qualified to decide—they have held only to honor and adorn The time has arrived when members of the profession should decline any positions, dependent for their permanency and stability upon popular clamor, political machination or personal malice. If the staffs of all beneficiary institutions so governed should be vacated by their medical officers, there would then perhaps be a change in this regard. Just so long as physicians allow themselves to be used, just so long will they be abused. H.

We have received a circular from a manufacturing drug-house in one of the Eastern cities, in which the writer, inclosing a certificate, from an analytical chemist, of the purity of his preparation, modestly requests us to publish, in our "editorial columns," selected passages from the certificate, "with such comments as you "[we] may feel free to make, compressed in a dozen or more lines, "for which we will thank you, and be pleased to forward you any "quantity of samples of articles you may name on our list. "Very respectfully," etc.

We once remember to have found among some old papers a copy-book written by one of a past generation. in which to the oft-repeated maxim, "Modesty is a quality that adorns a woman," the school-boy, weary of reiteration, had appended, "and damns a man." Surely the writer of the circular referred to must have arrived at the same conclusion. Should we comply with his request, we fear the editorial comments might not be such as would please him or increase the sale of his nostrums.

For such services, that is, the conversion of the JOURNAL into an advertising medium, we are to receive the thanks of the advertiser and as many samples of the advertised articles as we may desire. This we could not possibly afford. It is entirely too cheap. For a service altogether similar, we have been offered and refused five thousand dollars, and since then we have raised our price, which is equivalent to saying that the editorial pages of the JOURNAL are not for sale upon any terms. H.

For a long time past the amount of matter seeking publication in the columns of the JOURNAL has been in excess of its capacity. Almost every month valuable articles are returned to us by our publishers for want of space in which to print them, even after every expedient in the way of " solidification " has been resorted to to gain space. Some of this matter derives a large portion of its interest from its novelty, and would lose much, or all of its value, by delay. In order to accommodate still further our contributors, and its rapidly increasing subscription list, it has been decided to enlarge and otherwise improve the JOURNAL, from and after the close of the current year. This of course cannot be accomplished without increased expenditure of time, labor and money, for which there should necessarily be an adequate compensation. The subscription price of the JOURNAL will therefore be increased twenty-five per cent., that is, from three to four dollars per annum. We promise on our part a more than commensurate increase in the amount and value of its contents, and our publishers have given in advance, in the material, and in the typographical and mechanical excellence of the work, a guarantee of what they will do in the future. H.

---

***Who is a Regular Physician?***

This was the question to be decided upon definitely by the Medico-Historical Society during this month. Those present at the meetings are not likely to forget them very soon. They were reminded too strongly of the " *Parturiunt montes nascetur ridiculus*

*mus ;*" and a very small mouse indeed was the final production of so much labor of so many eminent gentlemen! The grand idea conceived by the Society, to arrest the growing evil of quackery, has come into flesh—as a "Medical Register." This child will be pelted by a few, hated by some, neglected by many, and good for nothing after all. The reasons for it are obvious, if we look at the scale used for gauging the standing of a physician.

On August 20th, a printed list of physicians "who are in good and regular standing" was submitted to the Medico-Historical Society for final action. At the head of this list we read the following resolution :

"Any physician who shall by any advertisement, placard, handbill, circular, pamphlet, book, card, or any public means, announce himself or herself as possessing unusual or peculiar skill in any branch of medicine or surgery, or who shall assume any title not specially granted by a regularly chartered college, shall not be eligible to membership in this Society ; and the name of such physician shall not be permitted to appear in the list of regular physicians published in the Chicago Medical Register ; *Provided*, that a physician may be permitted to affix or connect with his or her name the title or designation of a specialty when he or she shall have ceased to do any practice not pertaining to such specialty, and not otherwise."—*Resolution adopted by the Chicago Medico-Historical Society, August* 2, 1874.

Now, if the especial publication of this resolution (which, by the way, does not contain anything that was not said in the old code of ethics) should mean anything, it was to set forth the most salient reasons for stamping a physician *regular* or *irregular*. And, indeed, "Has he a regular diploma?" and "Does he not advertise?" these were the two vital questions of the evening. If the candidate complied with these requirements (as construed by this arbitrary board), he passed as *regular*. He might violate the universal code of ethics of gentlemanly conduct; he might publicly slander eminent members of the profession ; he might prescribe for a patient when he knew him to have another physician ; he might occasionally be "interviewed" on sanitary questions, or on certain famous legs, and let the reporter tell the public all about the superior attainments of the learned doctor ; he might have a card at the college building, announcing that the "Professor" treats certain diseases on certain days, gratis. All this is no advertising; it is done merely to enlighten the public. But woe for another who dared to enlighten the public, too! He was advertising, and ought to be stricken from the list. The definition of advertising is the most wonderful piece of rubber in existence; it can be stretched or contracted, just to suit the managers. For themselves they make liberal allowances, to let their lights shine before the public ; but the *dis inferiorum gentium* shall rest on their "professional dignity," and let nobody know, through the printing press or otherwise, anything of their skill.

Now, has it ever degraded anybody to announce what he is able to do, *as long as he can do it?* How, then, can a physician lower himself by announcing any peculiar skill, *as long as his actions prove him to possess it?* How should an honest advertisement injure the dignity of the profession? Because quacks fill the newspapers with boastful lies of wonderful cures, superior skill which they have not, therefore a regular physician must be silent. Because one man tells a lie, another man must not tell the truth. Is this logical reasoning? An educated doctor, who advertises honestly, is a quack no more than a quack who does not advertise is a regular physician.

You cannot eradicate quackery by such narrow-minded, unlogical restrictions as the code of ethics tries to force upon the regular physicians. And, because the Medical Register is founded on the dead letter of this code, it will be a failure; and, intended for distribution among physicians, it is a superfluous thing, because every physician, I trust, can soon enough find out for himself whether a certain doctor is an educated physician or a quack.

A "REGULAR."

---

### *Quarantine and the Public Health.*

OFFICE OF THE SUPERVISING SURGEON,
U. S. MARINE-HOSPITAL SERVICE,
*Treasury Department, September* 9, 1874.

SIR:

I am instructed by the honorable the Secretary of the Treasury to submit for your consideration the accompanying copy of Circular No. 90, relating to the Duties of U. S. Officers with reference to Quarantine and the Public Health, and to solicit from you such information and suggestions touching the subject-matter thereof as may conduce to the greatest efficiency and harmony of action between State and municipal health authorities on the one hand, and the officers of the National Government upon the other.

To prevent—so far as his official action in this connection may prevent—the interruption of commercial intercourse, with the consequent stagnation of business and loss of revenue which uniformly result from the access of an epidemic of contagious disease, is, obviously, the object of the Circular of the honorable the Secretary, charged, as he is, with the protection and improvement of the financial and commercial interests of the country.

The intimate correlation of these interests to the sanitary interests of the people—with the conservation of which you are charged—renders direct and intelligent co-operation between the agents of these interests of the first importance. Whatever information and suggestions to this end it may be in your power to furnish, are therefore desired, and will be made the basis of specific instructions and action whenever practicable.

Copies of your health laws and regulations—State, municipal, and port; of instructions to port physicians, pilots, boarding officers, and others; of descriptions of quarantine establishments, and of kindred published data, would be of immediate value, and may be forwarded direct to this office.

I have the honor to be, sir, your obedient servant,

JNO. M. WOODWORTH,
*Supervising Surgeon U. S. M. H. S.*

---

DUTIES OF UNITED STATES OFFICERS WITH REFERENCE TO QUARANTINE AND THE PUBLIC HEALTH.

1874—Department No. 90—Secretary's Office.

TREASURY DEPARTMENT, Sept. 8, 1874.

In the absence of uniformity in the regulations of quarantine upon the Atlantic and Gulf Coasts, it is desirable that the several officers specifically placed under the direction of the Treasury Department by the subjoined Section of The Revised Statutes of the United States, inform themselves fully as to the local health-laws, and the regulations based thereon and in force at their respective ports and stations, and strict compliance with such laws and prompt assistance in the enforcement of the same, when directed by competent authority, are hereby enjoined, in accordance with the provisions of the following:

"SECTION 4792. The quarantines and other restraints established by the health laws of any State, respecting any vessels arriving in, or bound to, any port or district thereof, shall be duly observed by

The officers of the customs revenue of the United States, by

The masters and crews of the several revenue-cutters, and by

The military officers commanding in any fort or station upon the sea-coast; and all such officers of the United States shall faithfully aid in the execution of such quarantines and health-laws, according to their respective powers, and within their respective precincts, and as they shall be directed from time to time by the Secretary of the Treasury." * * *

[*The Revised Statutes of the United States, p.* 890.]

Officers of the Customs Revenue are referred, in this connection, to Articles 294, 295, *General Regulations under the Customs and Navigation Laws of the United States*, 1874, and are requested to bring the same to the notice of the proper local health authorities, attracting attention especially to the second and third paragraphs of Article 294.

Officers in command of vessels of the Revenue Marine are instructed that Article 904 of said *Regulations* is held to include communication with infected vessels as well as ports; and, in order to render more efficient assistance to local authorities in the enforcement of quarantine laws, as therein directed, will take the necessary steps to advise such authorities of these instructions.

The co-operation of the military forces will be applied for only after exhausting the other powers and authorities herein mentioned; such application to be made to this Department, with a full statement of the facts for the information of the Honorable the Secretary of War.

Medical officers of the U. S. Marine-Hospital Service will govern their official action in consonance with this circular and the law as above cited; and will at all times assist as freely as practicable, not only other officers of the Government, but the quarantine authorities, in the protection of the public health against the introduction of contagious diseases. In furtherance of this end, they are instructed to communicate to the Supervising Surgeon, from time to time, such information and suggestions as will enable that officer to frame needful regulations, and to take intelligent action in cases of emergency.

B. H. BRISTOW,
*Secretary of the Treasury.*

